# Supplementary material for: Effectiveness of Mindfulness-Based Interventions with Respect to Psychological and Biomedical Outcomes in Young People with Type 1 Diabetes: A Systematic Review
Source: Healthcare (Basel). 2024 Sep 19;12(18):1876. doi: 10.3390/healthcare12181876 (PMC11430895; doi:10.3390/healthcare12181876)
Supplement: Supplementary file 1 [file healthcare-12-01876-s001.zip › Table S2 Search Strategies.pdf]

Table S2 Search Strategies

Data sources	Search strategies
WOS Core collection	ALL=((Adolescen* OR Teen* OR Youth* OR “Young people”) AND (diabetes OR T1D* OR idd*) AND (Mindful* OR Mind-Body* OR MBI OR MBSR OR MBCT OR MB-EAT OR Meditation*))
MEDLINE	(Adolescen* OR Teen* OR Youth* OR "Young people") AND (diabetes OR T1D* OR idd*) AND (Mindful* OR Mind-Body* OR MBI OR MBSR OR MBCT OR MB-EAT OR Meditation*)
Scielo (via WOS)	ALL=((Adolescen* OR Teen* OR Youth* OR “Young people”) AND (diabetes OR T1D* OR idd*) AND (Mindful* OR Mind-Body* OR MBI OR MBSR OR MBCT OR MB-EAT OR Meditation*))
SCOPUS	TITLE-ABS-KEY((Adolescen* OR Teen* OR Youth* OR "Young people") AND (diabetes OR T1D* OR idd*) AND (Mindful* OR Mind-Body* OR MBI OR MBSR OR MBCT OR MB-EAT OR Meditation*))
PsycINFO	ab(Adolescen* OR Teen* OR Youth* OR “Young people”) AND ab(diabetes OR T1D* OR idd*) AND ab(Mindful* OR Mind-Body* OR MBI OR MBSR OR MBCT OR MB-EAT OR Meditation*)
Cochrane	(Adolescen* OR Teen* OR Youth* OR “Young people”) AND (diabetes OR T1D* OR idd*) AND (Mindful* OR Mind-Body* OR MBI OR MBSR OR MBCT OR MB-EAT OR Meditation*) in Title Abstract Keyword
